# Patient-Reported Adherence, Knowledge and Perceived Outcomes After Specialist Nursing Education in a Multidisciplinary Headache Unit: A Cross-Sectional Observational Study

**DOI:** 10.3390/nursrep16100359

**Published:** 2026-10-10

**Authors:** María José Ruiz-Castrillo, Leonardo Portocarrero-Sánchez, Lucía Zaballa-Pérez, Alejandro Sánchez-Huertas, Javier Díaz-de-Terán

**Affiliations:** 1 Headache and Neuropathic Pain Unit, Department of Neurology, Hospital Universitario La Paz, 28046 Madrid, Spain; coteruizca@gmail.com (M.J.R.-C.); leonardo9493@gmail.com (L.P.-S.); luciazaballaperez@gmail.com (L.Z.-P.); alejandrosanchezhuertas@gmail.com (A.S.-H.); 2 Hospital La Paz Institute for Health Research (IdiPAZ), Hospital Universitario La Paz, Universidad Autónoma de Madrid, 28046 Madrid, Spain

**Keywords:** migraine disorders, nursing, patient education, medication adherence, quality of life, headache disorders, calcitonin gene-related peptide

## Abstract

**Background/Objectives:** Migraine is a leading cause of disability worldwide. Despite advances such as calcitonin gene-related peptide (CGRP)-targeted therapies, suboptimal treatment adherence, limited health literacy, and weak self-management remain common, and patient-reported data on the contribution of specialist headache nurses within European tertiary units are scarce. This study describes patient-reported adherence to acute treatment and to lifestyle recommendations, self-perceived knowledge, and perceived quality of life in adults with migraine who had attended a structured, nurse-led educational consultation in a tertiary multidisciplinary headache unit. **Methods:** This cross-sectional descriptive study included 90 adults (≥18 years) with migraine diagnosed according to the International Classification of Headache Disorders, third edition (ICHD-3), who were starting treatment with an anti-CGRP monoclonal antibody and consecutively attended a specialist nursing consultation; patients with documented cognitive impairment or without written informed consent were excluded. Between January and December 2021, participants completed, on a single occasion after the consultation, an electronic, structured, piloted ad hoc survey covering knowledge (with pre-consultation knowledge rated retrospectively), adherence to pharmacological and lifestyle recommendations, perceived changes in attack frequency and intensity, and self-reported quality of life. Data were analysed descriptively, with 95% confidence intervals (CIs) for proportions and without formal hypothesis testing. **Results:** Participants were mostly women (67.8%), with a mean age of 43.1 ± 10.7 years. Overall, 74.4% (95% CI 64.6–82.3) reported always following acute-treatment instructions and 66.7% (56.4–75.5) always following lifestyle recommendations; 68.9% (58.7–77.5) felt the education had improved their understanding of migraine and their quality of life, and 67.8% (57.6–76.5) perceived fewer attacks. Self-rated adequate knowledge was higher at the time of the survey than retrospectively recalled for the pre-consultation period in all five domains. The main barriers were limited prior knowledge and difficulty maintaining a headache diary. **Conclusions:** Most patients who had received structured nurse-led education reported good adherence and perceived benefits in their understanding of migraine and quality of life. Given the cross-sectional design, the retrospective assessment of prior knowledge, the use of a non-validated questionnaire, and the absence of a control group, these findings cannot be attributed to the consultation itself, and perceived changes in attack frequency cannot be separated from the effect of the newly started anti-CGRP treatment; prospective, controlled studies using validated outcome measures are needed. This study was not registered.

## 1. Introduction

Migraine is one of the most common and disabling neurological disorders. According to the Global Burden of Disease 2023 study, headache disorders affected 2.9 billion people worldwide in 2023, with an age-standardised prevalence of 34.6%; migraine accounted for about 90% of the years lived with disability attributed to headache disorders, and this burden was more than twice as high in females as in males [1]. Age-standardised prevalence has remained stable over three decades [1], and a 30-year trend analysis projecting forward to 2050 points to further increases in the number of people affected and in disability [2]. Migraine is the leading cause of disability-adjusted life-years (DALYs) in people under 50 and the main cause of years lived with disability among women of reproductive and working age (20–49 years); for this age group, the female-to-male ratio is greater than 2:1 [3,4]. Its consequences reach well beyond the clinic, affecting work, relationships, and mental health. The burden is not limited to the pain of individual attacks. Between attacks, many patients continue to experience anxiety, social withdrawal, cognitive difficulty, disturbed sleep, and mood disorders [5]. Direct costs such as emergency visits, specialist appointments, and medication, together with the indirect costs of lost productivity, make migraine an expensive and frequently underestimated public health problem [6]. A substantial proportion of people with migraine never receive an appropriately prescribed acute treatment and instead rely on over-the-counter analgesics taken without medical supervision. Used this way, acute medication can itself drive medication-overuse headache (headache on 15 or more days per month with acute medication overuse for at least three months) and push episodic migraine toward a chronic course, an outcome that is largely preventable with early education and structured follow-up [7,8]. In GBD 2023, medication-overuse headache accounted for 14–23% of the years lived with disability attributed to migraine, a share of the burden that the authors considered largely avoidable through education on medication use [1].

The past decade has reshaped migraine prevention with the approval of monoclonal antibodies against CGRP or its receptor and gepants. CGRP is a vasoactive neuropeptide released from trigeminal sensory endings during attacks; when its levels rise, both during and between attacks, it promotes neurogenic inflammation of meningeal vessels and sensitises trigeminal pathways peripherally and centrally, amplifying and sustaining pain [9,10]. Four monoclonal antibodies are now approved for prevention, including erenumab, which targets the receptor, and fremanezumab, galcanezumab, and eptinezumab, which target the ligand [11]. Pivotal trials and later real-world studies have shown consistent efficacy in both episodic and chronic migraine, with the proportion of patients achieving at least a 50% reduction in monthly migraine days rising over time and tolerability that generally compares favourably with older preventives [12,13]. Real-world data also show gains in treatment satisfaction and quality of life in patients with resistant migraine [14]. These benefits matter at a societal level too, given that migraine is a leading source of lost working days and reduced workplace productivity [15].

Medication alone, though, does not deliver the full benefit. Patients still need to know how to use their treatment, how to spot their triggers, which lifestyle factors to work on, and how to keep a headache diary, all of which help get the most out of treatment and lower the risk of chronification [7,16]. Sleep deserves particular attention in this respect. Poor subjective sleep quality is common in people with headache and orofacial pain and appears closely linked to depression, anxiety, and stress [17], and polysomnographic data have associated chronic migraine with reduced REM sleep [18]. Because sleep disturbances may affect daily functioning, quality of life, and the ability to follow treatment and lifestyle recommendations, sleep is one of the lifestyle factors routinely addressed in patient education. As migraine has come to be understood as a multidimensional condition, dedicated multidisciplinary headache units have grown up around it, and specialist nurses have taken on a steadily larger role within them [19,20,21]. With anti-CGRP therapy in particular, the nurse’s input into injection training, watching for adverse effects, and counselling patients on what to expect is often what keeps them on treatment long enough to benefit [13].

A European three-round e-Delphi study published in 2024 reached consensus on five core nursing competencies in headache care: taking the patient history, delivering individualised education, taking part in follow-up and telephone triage, monitoring adverse effects, and running group sessions [22]. These recommendations give structure to a role that has long varied from one European centre to another. In parallel, systematic reviews of nurse-led care in chronic disease have shown that structured, face-to-face nursing education improves medication adherence across a range of conditions, with individualised counselling and motivational interviewing among the most effective approaches [23,24].

Despite this growing evidence in chronic disease more broadly, good-quality data on how patients themselves experience structured nursing consultations within headache units are still scarce [25,26]. The present study addresses that gap by describing patient-reported adherence to acute treatment and to lifestyle recommendations, self-perceived knowledge, and perceived quality of life among patients with migraine starting anti-CGRP monoclonal antibodies who had attended a structured specialist nursing consultation in a tertiary referral headache unit.

## 2. Materials and Methods

### 2.1. Study Design and Setting

This was a cross-sectional descriptive study at the headache unit of a tertiary referral centre (Hospital Universitario La Paz, Madrid, Spain). The unit is a dedicated multidisciplinary service caring for patients with primary and secondary headache disorders, with access to newer preventive treatments such as anti-CGRP monoclonal antibodies. Data were collected between January and December 2021. All data were obtained from a single administration of a self-completed survey after the nursing consultation; no prospective baseline measurement was taken, and there was no control group. The before-and-after knowledge data reported in Section 3.5 therefore reflect a retrospective self-assessment, in which patients rated, within the same survey, their knowledge before the consultation and at the time of completion. The study is reported in accordance with the STROBE (Strengthening the Reporting of Observational Studies in Epidemiology) statement for cross-sectional studies.

### 2.2. Participants

Participants were adults (≥18 years) with migraine diagnosed according to ICHD-3 criteria who were starting treatment with an anti-CGRP monoclonal antibody and had been referred to the specialist nursing consultation for individualised education on the new treatment, acute management, and lifestyle. We excluded patients with documented cognitive impairment and those who did not give written informed consent. Recruitment was consecutive: all patients who attended the nursing consultation during the study period and met these criteria were informed about the study, and those who agreed to take part and gave written consent were included. Of the 100 patients invited, 90 agreed to participate (participation rate, 90%). No formal sample size calculation was performed; the sample comprised all eligible patients who agreed to take part during the 12-month study period. With 90 participants, the 95% CI around any proportion has a maximum half-width of about ±10 percentage points, which we considered acceptable for a descriptive study.

### 2.3. Nursing Intervention

The consultation was a structured, individualised session led by a specialist nurse with advanced training in neurology and headache, which patients attended after their visit with the neurologist in the headache unit. It covered education about migraine, including its pathophysiology and the rationale for the prescribed treatment; non-pharmacological treatment, with the identification of individual triggers and modifiable lifestyle factors such as regular sleep, adequate hydration, aerobic exercise, stress management, and regular meals, and how to keep a headache diary; the optimisation of acute treatment, including the correct use of triptans and NSAIDs, with attention to timing, dosing, and avoiding overuse; and the detection of medication overuse. Because all participants were starting an anti-CGRP monoclonal antibody, every session also covered self-injection technique, rotating injection sites, recognising possible adverse effects, and what to expect in terms of onset and response [11,13]. Time was also set aside in every session to resolve all of the patient’s questions. All consultations were delivered by the same specialist nurse and lasted approximately 30 min. The content followed a common structure for all patients, while leaving room to adapt it to each patient’s needs. At the end of the session, patients received a written sheet of recommendations prepared by the headache unit team. After the consultation, patients were followed up by both the headache neurologist and the specialist nurse, as is standard for all patients attended in the unit.

### 2.4. Data Collection Instrument

Data were collected with a structured ad hoc survey developed by the research team and reviewed by a panel of three experts in headache: two neurologists specialising in headache, and one nurse specialising in headache care. The instrument was piloted in 50 patients before use, after which two items that patients found difficult to understand were reworded. Beyond this expert review of content and the pilot assessment of comprehensibility, no formal psychometric evaluation was carried out: reliability (internal consistency or test–retest) and construct validity were not assessed, and most constructs were measured with single items. The final version comprised 25 items grouped into six sections: knowledge in five domains (trigger identification, acute medication use and timing, lifestyle modification, headache diary completion, and prevention of medication-overuse headache), each rated for the period before the consultation (retrospectively) and at the time of the survey (items 1–5); adherence to the nurse’s recommendations on lifestyle, acute treatment, and headache diary completion (items 6–8); anti-CGRP treatment, covering self-injection technique, adverse effects, expected time to effect, and adherence to the administration schedule (items 9–12); perceived changes in attack frequency and intensity, understanding of migraine and quality of life, confidence in managing the condition, and willingness to recommend the consultation (items 13–17); assessment of the consultation, covering clarity of the information, time available, resolution of questions, usefulness of the written recommendations, and knowing how to contact the unit (items 18–22); and barriers to following the recommendations, overall satisfaction on a 0–10 scale, and suggestions for improvement (items 23–25). Sociodemographic and clinical data were obtained from the clinical records. This paper reports 14 of the 25 items (items 1–8, 13–17, and 23), covering knowledge, adherence, perceived outcomes, and barriers. These outcomes were selected by consensus among the members of the headache unit on the basis of two criteria: their direct relevance to the objectives of the study, and completeness of responses so that only items answered by all 90 participants were analysed. The remaining items (anti-CGRP treatment, assessment of the consultation, overall satisfaction, and suggestions) are not analysed in this paper; several of them had incomplete responses. Because completeness could only be established once the data had been collected, this selection was not prospectively registered. The survey was completed electronically on a single occasion: it was sent to each patient by e-mail a median of 120 days (interquartile range [IQR] 90–140) after the nursing consultation and completed at home. To limit social desirability bias, patients answered it without the nurse who had delivered the consultation being present. The full questionnaire (original Spanish version and English translation) is provided as Supplementary File S2.

### 2.5. Outcome Definitions

Adherence to acute treatment and adherence to lifestyle recommendations were each assessed with a single item rated on a four-point frequency scale: always; usually (more than 50% of the time); sometimes (50% of the time or less); and never. Good adherence was defined as a response of “always”. For acute treatment, the item referred to following the nurse’s instructions on timing, dosing, and avoiding overuse; for lifestyle, it referred to regular sleep, adequate hydration, aerobic exercise, and stress management. Perceived change in attack frequency, perceived improvement in understanding of migraine and quality of life, confidence in managing the condition, and willingness to recommend the consultation were each assessed with a single item, analysed as yes/agree versus no/disagree. Quality of life was therefore captured as a single global self-perception rather than with a validated multi-item instrument such as the Migraine-Specific Quality of Life Questionnaire (MSQ) [27]. Knowledge was assessed in five domains (trigger identification, acute medication use and timing, lifestyle modification, headache diary completion, and prevention of medication-overuse headache). For each domain, patients rated their knowledge before the consultation and at the time of the survey, and knowledge in each domain was rated on a four-point scale (none, a little, quite a lot, a lot), and a rating of “quite a lot” or “a lot” was classified as adequate knowledge. The same criterion was applied to all five domains and was agreed by the headache team (neurologists and specialist nurse) before data collection.

### 2.6. Ethical Considerations

The study was approved by the Clinical Research Ethics Committee (Comité de Ética de la Investigación con Medicamentos, CEIm) of Hospital Universitario La Paz, Madrid, Spain (protocol code PI4356; date of approval: 11 June 2020), and conducted in accordance with the Declaration of Helsinki and the applicable legislation on biomedical research and personal data protection. All participants gave written informed consent before inclusion.

### 2.7. Statistical Analysis

Analyses were carried out in IBM SPSS Statistics (version 23.0; IBM Corp., Armonk, NY, USA). Categorical variables are reported as absolute frequencies and percentages, and continuous variables as mean ± standard deviation and range. Given the descriptive, exploratory design, no formal hypothesis testing was performed. Proportions for the main outcomes are presented with 95% confidence intervals (CIs) calculated with the Wilson score method. Because pre-consultation knowledge was rated retrospectively in the same survey as current knowledge, the two ratings were not compared statistically and are presented descriptively only. There were no missing data in the items analysed, and all percentages are calculated over the 90 participants.

## 3. Results

### 3.1. Sample Characteristics

Ninety patients were included. Most were women (61; 67.8%), and the mean age was 43.1 ± 10.7 years (range 18–72). By design, all were starting treatment with an anti-CGRP monoclonal antibody: erenumab in 30 (33.3%), galcanezumab in 21 (23.3%), and fremanezumab in 39 (43.3%). Their clinical characteristics are summarised in Table 1. Fifty-six patients (62.2%) had migraine without aura and 34 (37.8%) migraine with aura; 70 (77.8%) had chronic and 20 (22.2%) episodic migraine, with a mean disease duration of 18.0 ± 6.3 years and 23 ± 5 monthly headache days. Medication overuse was present in 71 patients (78.9%) at the time of the nursing consultation, and patients had received a mean of 7.3 ± 2.7 previous preventive treatments, including oral preventives. Concomitant oral preventive treatment was maintained in 36 patients (40.0%) and onabotulinumtoxinA in 14 (15.6%). The most frequent comorbidities were anxiety and/or depression (70; 77.8%) and sleep disorders (55; 61.1%). The survey was completed a median of 120 days (IQR 90–140) after the nursing consultation.

### 3.2. Adherence to Lifestyle Recommendations

**Figure 1:**
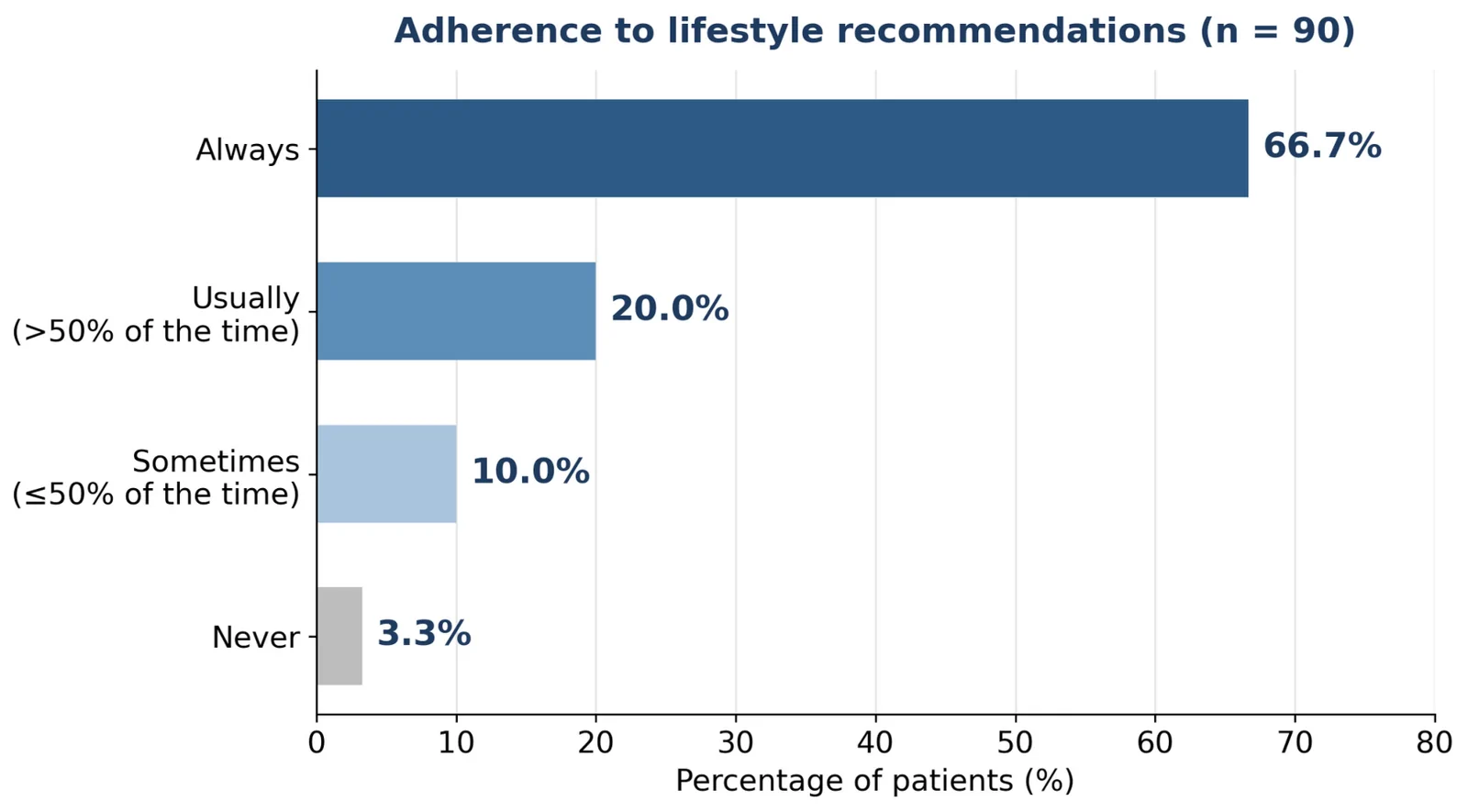
Distribution of patient-reported adherence to lifestyle recommendations following specialist nursing consultation (*n* = 90). Percentages indicate frequency of adherence across four response categories.

Sixty patients (66.7%; 95% CI 56.4–75.5) reported always following the lifestyle advice they had been given, covering regular sleep, adequate hydration, aerobic exercise, and stress management (Figure 1). A further 18 (20.0%) reported following it usually, 9 (10.0%) sometimes, and 3 (3.3%) never.

### 3.3. Adherence to Acute Pharmacological Treatment

**Figure 2:**
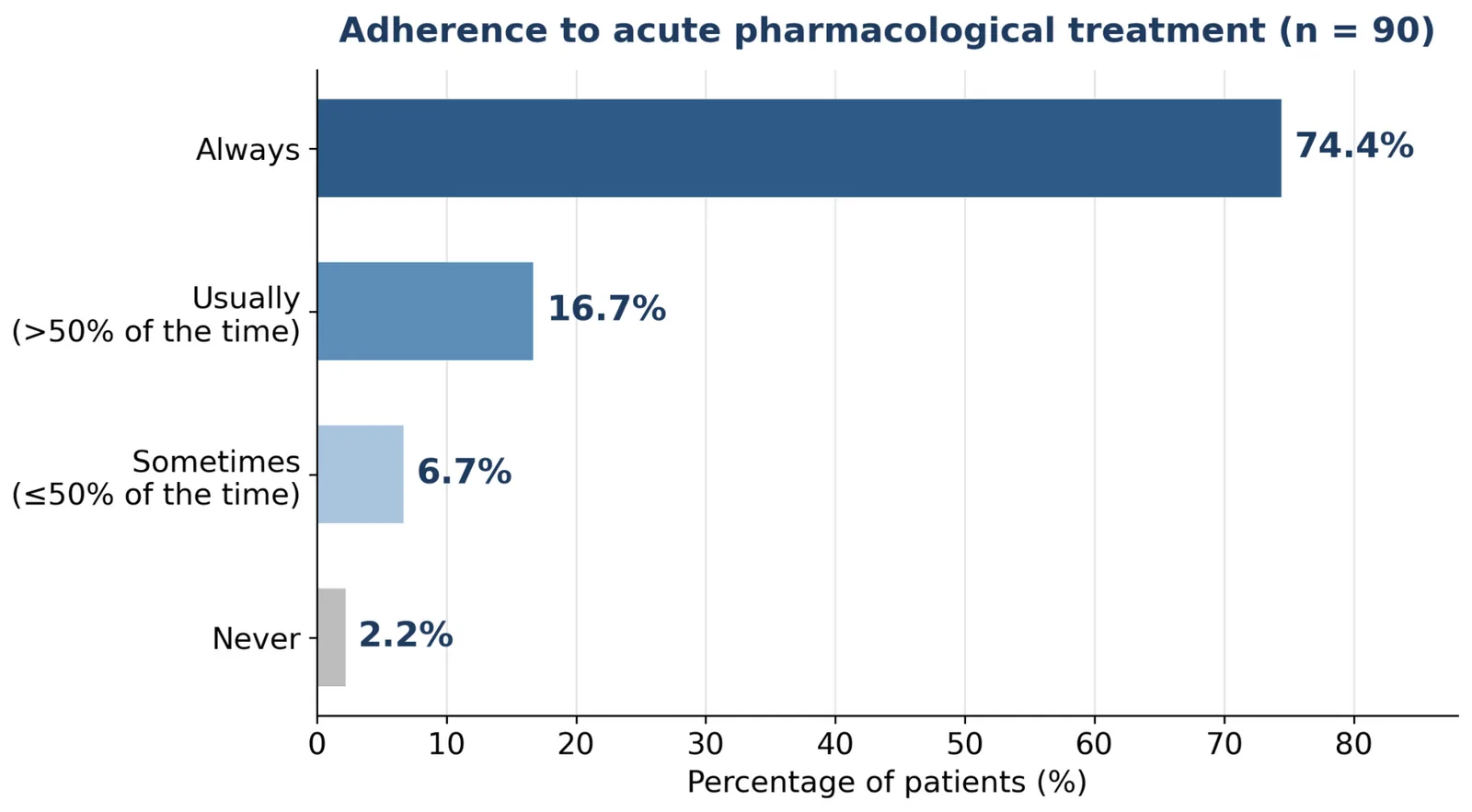
Distribution of patient-reported adherence to acute pharmacological treatment following specialist nursing consultation (*n* = 90). Percentages indicate frequency of correct medication use across four response categories.

Sixty-seven patients (74.4%; 95% CI 64.6–82.3) reported always following the nurse’s instructions on timing, dosing, and avoiding overuse of acute medication, which met the definition of good adherence (Figure 2). A further 15 (16.7%) reported doing so usually, 6 (6.7%) sometimes, and 2 (2.2%) never.

### 3.4. Perceived Quality of Life, Attack Frequency, and Self-Management

**Figure 3:**
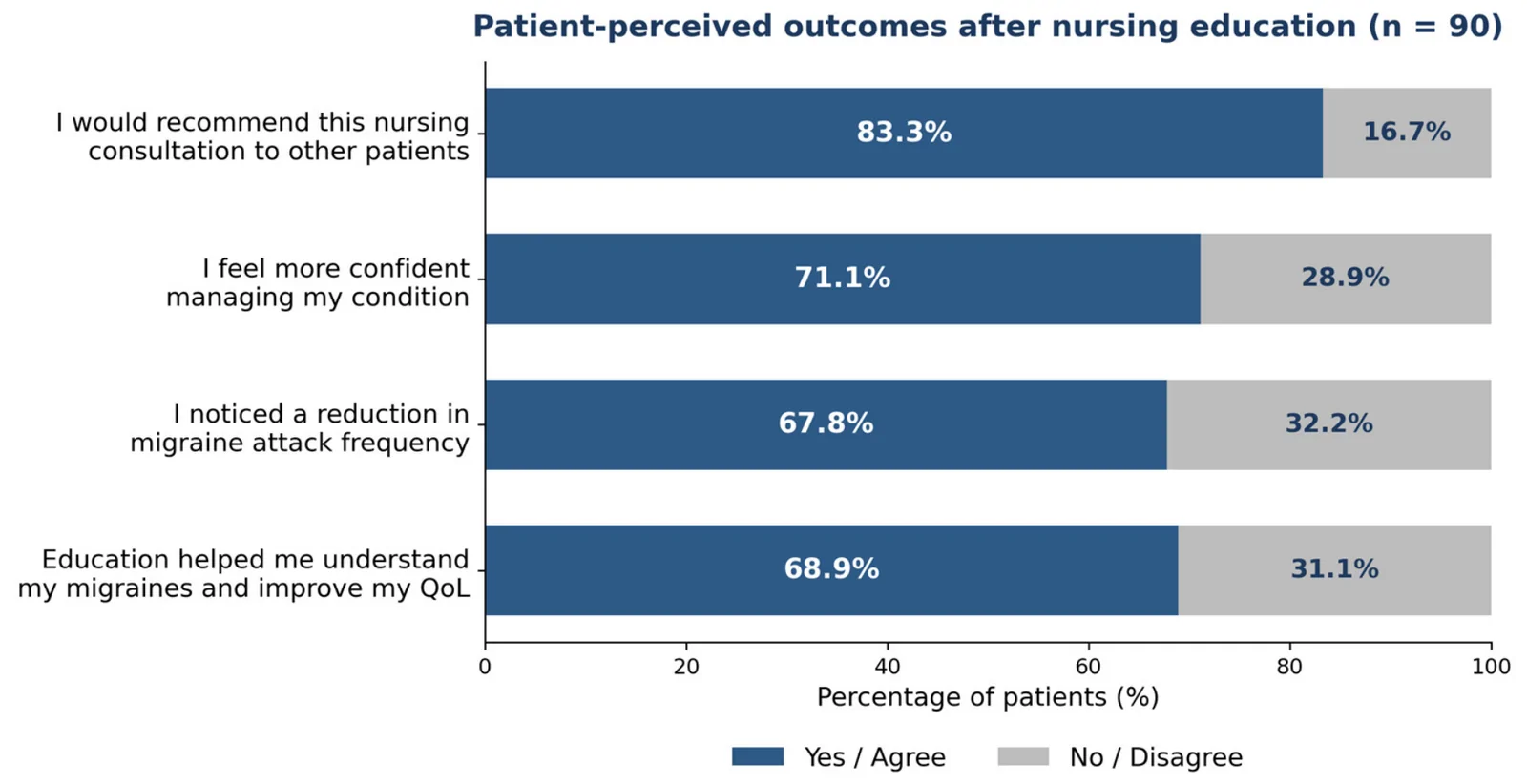
Patient-perceived outcomes after specialist nursing education across four domains (*n* = 90). Bars represent the proportion of patients answering yes/agree versus no/disagree to each single survey item.

At the time of the survey, 62 patients (68.9%; 95% CI 58.7–77.5) agreed that the education had helped them understand their condition better and had improved their quality of life, and 61 (67.8%; 95% CI 57.6–76.5) reported perceiving fewer attacks since putting the nurse’s advice into practice. In addition, 64 (71.1%; 95% CI 61.0–79.5) felt more confident managing their condition, and 75 (83.3%; 95% CI 74.3–89.6) would recommend the consultation to other patients (Figure 3).

### 3.5. Self-Reported Knowledge

**Figure 4:**
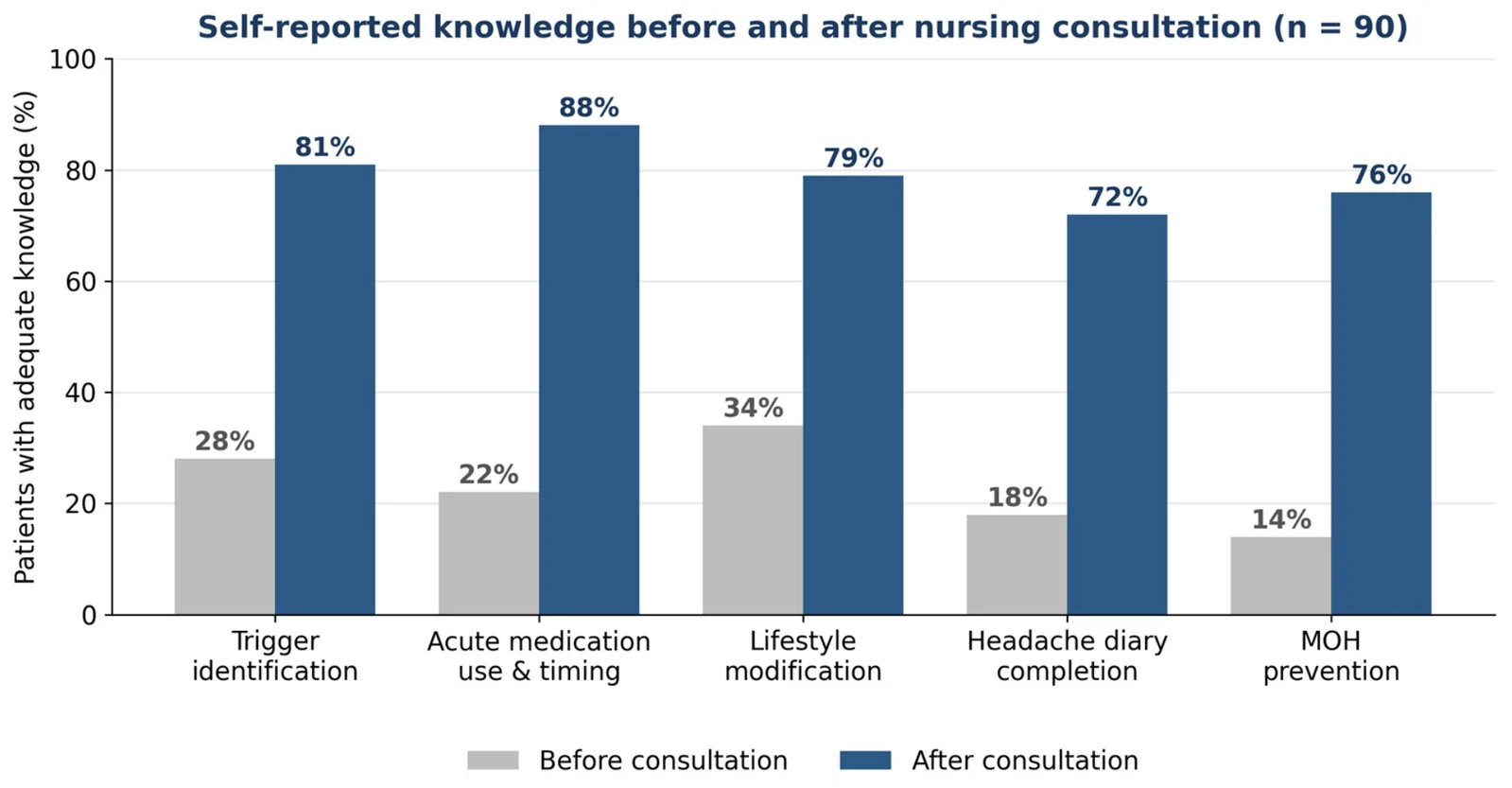
Self-reported patient knowledge before and after specialist nursing consultation across five clinical domains (*n* = 90). Both ratings were collected in the same survey after the consultation; pre-consultation knowledge was rated retrospectively. Bars represent the proportion of patients with adequate self-reported knowledge for each period, defined a priori as a rating of “quite a lot” or “a lot” on a four-point scale.

In the retrospective self-assessment, the proportion of patients rating their knowledge as adequate was lower for the period before the consultation than at the time of the survey in all five domains: trigger identification (28% vs. 81%), acute medication use and timing (22% vs. 88%), lifestyle modification (34% vs. 79%), headache diary completion (18% vs. 72%), and prevention of medication-overuse headache (14% vs. 76%) (Figure 4). Both ratings were given in the same survey, after the consultation.

### 3.6. Barriers Identified

The main barriers patients described were limited knowledge of migraine and its management before the consultation, and difficulty keeping the headache diary up to date. A smaller number mentioned time pressure and competing demands at work.

## 4. Discussion

In this cohort of patients starting anti-CGRP monoclonal antibodies who had attended a structured specialist nursing consultation, most reported good adherence to acute treatment and to lifestyle advice, and around two-thirds felt that the education had improved their understanding of migraine and their quality of life. These are patient-reported observations collected in a single survey after the consultation, and we have tried to read them as such: they describe how patients see their own behaviour and outcomes after nursing education, not an effect that can be attributed to the consultation itself. They were also collected with an ad hoc, non-validated questionnaire in which most outcomes rely on a single item, which further limits the strength of any conclusion that can be drawn from them. The sample, mostly women of working age, reflects the known epidemiology of migraine [3,4]. With these caveats, the findings are consistent with a picture that has been building for several years now, in which the headache nurse is no longer a peripheral figure but a core part of how these patients are cared for [22,25].

The thread running through all of this is simple: you cannot manage migraine well with a prescription alone. Patients who understand their condition, what sets it off, and why their treatment works the way it does, use their acute medication better and are less likely to slip into the overuse that drives chronification [7,8]. When neurologists have only a few minutes per patient, the nursing consultation is usually where this teaching actually happens [19,21].

The proportion of patients reporting good adherence to acute treatment in our cohort (74.4%) appears higher than what is often described in routine care, where patients often use their medication poorly or fall back on whatever analgesic is to hand, which limits benefit and raises the risk of medication overuse [7,8,16]. This comparison should be made cautiously, since our data are self-reported, definitions of adherence differ between studies, and we had no comparison group. Our findings are in line with Kindelan-Calvo and colleagues, whose meta-analysis found that structured education improves both adherence and patients’ confidence in managing their migraine [16]. They also echo the older nurse-based model of Cady and colleagues, back in 2008, which improved knowledge, medication use, and disability scores [28]. Two more recent reviews say much the same from a wider angle: Berardinelli and colleagues found that face-to-face, nurse-led education improves adherence across chronic diseases, motivational interviewing especially [23], and Ruksakulpiwat and colleagues saw gains in adherence, function, and quality of life among patients with multimorbidity [24].

The perceived improvements in quality of life (68.9%) and attack frequency (67.8%) need to be read with particular caution. Each was captured by a single global item, and a perceived reduction in attacks may reflect the natural fluctuation of migraine, regression to the mean, and, above all, the fact that every patient had just started an anti-CGRP monoclonal antibody, an effective preventive treatment whose effect our design cannot separate from that of the education. Reductions in disability have been reported after structured education in randomised and comparative studies [16,28], and Baker and Fatoye, reviewing qualitative research in chronic obstructive pulmonary disease, found that patients describe nurse-led self-management as leaving them more knowledgeable, more confident, and generally better in themselves [26]. Quantifying any such change in migraine requires validated measures (MIDAS, HIT-6, and the MSQ), and future studies should be built around them rather than around home-made surveys [27].

The knowledge data call for a similar caveat. Because patients rated their prior knowledge retrospectively, after the consultation, they may have judged what they knew beforehand against what they had just learnt, a form of response shift that tends to exaggerate the apparent gain. We therefore read Figure 4 as patients’ perception of what they took away from the consultation, not as a measured change in knowledge.

If anything, the shift toward anti-CGRP prevention raises the stakes for nursing education rather than lowering them. Now that erenumab, fremanezumab, galcanezumab, and eptinezumab are considered a first-line preventive option [29], someone has to show patients how to inject, set honest expectations about when the drug will start working, flag the side effects worth watching for, and explain why it is worth staying the course over several cycles [11,13]. Response rates keep climbing the longer patients stay on treatment, and persistence depends on factors that structured support may help to address [13,14]. And because migraine is one of the biggest causes of lost working days [15], keeping patients on effective prevention pays off well beyond the clinic. All our patients were starting one of these antibodies, so the consultation we describe is a realistic model of nursing support at treatment initiation; however, we did not measure persistence or long-term outcomes, and whether this support influences them deserves specific study.

The European e-Delphi consensus from Rasmussen and colleagues (education, history-taking, follow-up, monitoring side effects, and running group sessions) reads almost like a job description for the role we are describing [22]. What our data add is a description of how patients in a Spanish tertiary unit report their adherence and perceived benefit after a consultation built around these competencies, within the European literature that remains surprisingly thin [25].

The barriers our patients named, not knowing much about migraine beforehand, and struggling to keep the diary going, are the same ones that turn up across the headache literature [5,30]. This suggests that nursing support may be most useful when it is offered early and is easy to reach. It also leaves an obvious opening for technology: a well-designed headache-diary app, or a simple messaging line to the nurse, might take much of the friction out of self-monitoring between visits. Sleep, although covered in the lifestyle education, was not specifically assessed in our survey, even though 61.1% of our patients had a documented sleep disorder; given its close relationship with headache and its potential influence on adherence and quality of life [17,18], sleep quality should be measured in future studies of nursing education.

### Limitations

This study has several limitations, and most of them stem from its design. First, it is a cross-sectional study with a single post-consultation survey and no control group, so the outcomes we describe cannot be attributed to the nursing consultation. In particular, all patients were starting an anti-CGRP monoclonal antibody, so perceived reductions in attack frequency and improvements in quality of life cannot be separated from the effect of the drug; neurologist follow-up and other components of care may also have contributed. Second, pre-consultation knowledge was rated retrospectively in the same survey, which exposes it to recall bias and to response shift and may overstate the perceived change; for this reason, the before-and-after ratings were not compared statistically. Third, the survey was completed a median of 120 days (IQR 90–140) after the consultation, so perceived attack frequency and sustained lifestyle adherence cover different periods for different patients and may be influenced by the natural fluctuation of migraine and by regression to the mean. Fourth, no sample size calculation was performed, only patients who agreed to take part were included, and those who were more satisfied or more adherent may have been more willing to do so; moreover, although the survey was completed at home and without the nurse present, it was sent by the unit that provided their care, which may still have favoured socially desirable answers. Fifth, all outcomes were self-reported with an ad hoc instrument that, although reviewed by headache specialists and piloted, has not been formally validated, and its reliability and validity were not assessed; several constructs, including quality of life, were captured by single items, and one item combined understanding of migraine and quality of life, so the two cannot be separated; moreover, adequate knowledge was defined by a cut-off on a self-rated scale, agreed within the team before data collection, rather than by an objective knowledge test. Only 14 of the 25 questionnaire items are reported; they were selected for their relevance to the study objectives and the completeness of responses, but this selection was not prospectively registered, and a risk of selective reporting cannot be excluded. We did not use validated measures such as MIDAS, HIT-6, or the MSQ [27], nor did we assess sleep quality, which may influence both adherence and quality of life [17,18]. Finally, this was a single-centre study of 90 patients starting anti-CGRP treatment, a population with high-frequency migraine (77.8% chronic migraine and 78.9% medication overuse), which limits generalisability to the wider population of people with migraine. A prospective design with baseline and follow-up measurements, validated instruments, and a concurrent control group receiving standard neurological care would be needed to estimate the effect of the nursing consultation, and building and validating a nursing-outcome instrument for headache care is a job still waiting to be done.

## 5. Conclusions

In this cross-sectional study, most patients starting anti-CGRP monoclonal antibodies who had attended a structured specialist nursing consultation in a multidisciplinary headache unit reported good adherence to acute treatment and to lifestyle recommendations, and about two-thirds felt that the education had improved their understanding of migraine and their quality of life. Because these are self-reported data from a single survey, collected with an ad hoc, non-validated instrument in which most outcomes rely on a single item and without a control group, they describe patients’ experience after nursing education rather than demonstrating its effect.

We are arguably living through the best period in the history of headache medicine. Targeted treatments once thought unimaginable, the anti-CGRP monoclonal antibodies and gepants, have already changed patients’ lives, and agents directed at new targets such as PACAP may change them further. Even so, previous studies and expert consensus indicate that structured patient education remains a key component of migraine care [16,22], and nursing within dedicated headache units is well placed to deliver it.

With anti-CGRP antibodies now considered a first-line preventive option [29], the nursing role in injection training, setting expectations, and supporting adherence is likely to become more relevant. Whether structured nursing input improves persistence and long-term outcomes with these treatments is a question our study was not designed to answer, and one that deserves specific investigation.

Spanish clinical practice guidelines already count the multidisciplinary team, nursing included, as a pillar of modern headache management [31], and our findings are consistent with that position. Establishing the contribution of structured nursing consultations will require prospective, multicentre, controlled studies built on validated instruments—MIDAS, HIT-6, and the MSQ—with a specific eye on nursing’s role in anti-CGRP adherence and long-term outcomes. A validated, standard nursing-outcome measure for headache care would be a real contribution in its own right.

## Figures and Tables

**Table 1:** Demographic and clinical characteristics of the participants.

Characteristic	*n* = 90
Age, years, mean ± SD (range)	43.1 ± 10.7 (18–72)
Female sex, *n* (%)	61 (67.8)
**Migraine type and course, *n* (%)**	
Without aura	56 (62.2)
With aura	34 (37.8)
Episodic migraine	20 (22.2)
Chronic migraine	70 (77.8)
Disease duration, years, mean ± SD	18.0 ± 6.3
Monthly headache days, mean ± SD	23 ± 5
Medication overuse at consultation, *n* (%)	71 (78.9)
**Preventive treatment**	
Previous preventive treatments (including oral), mean ± SD	7.3 ± 2.7
Anti-CGRP monoclonal antibody started, *n* (%)	90 (100)
Erenumab	30 (33.3)
Galcanezumab	21 (23.3)
Fremanezumab	39 (43.3)
Concomitant oral preventive, *n* (%)	36 (40.0)
Concomitant onabotulinumtoxinA, *n* (%)	14 (15.6)
**Comorbidities, *n* (%)**	
Anxiety and/or depression	70 (77.8)
Sleep disorders	55 (61.1)
Time from consultation to survey, days, median (IQR)	120 (90–140)

CGRP, calcitonin gene-related peptide; IQR, interquartile range; SD, standard deviation.

## Data Availability

The data presented in this study are available on request from the corresponding author due to restrictions protecting patient confidentiality.

## Supplementary Materials

The following supporting information can be downloaded at: https://www.mdpi.com/article/10.3390/nursrep16100359/s1. The following files are provided: File S1: STROBE checklist for cross-sectional studies; File S2: Study questionnaire (original Spanish version and English translation).

## Public Involvement Statement

No public involvement in any aspect of this research.

## Guidelines and Standards Statement

This manuscript was drafted against the STROBE (Strengthening the Reporting of Observational Studies in Epidemiology) statement for cross-sectional studies.

## Use of Artificial Intelligence

During the preparation of this manuscript, the authors used Claude (Opus 5.5, Anthropic, San Francisco, CA, USA), a generative AI tool, to assist with language editing and restructuring of the text, including revisions made in response to the reviewers’ comments. This tool was not used for study design, data collection, data analysis, or interpretation of the results, nor for generating the scientific content of the manuscript. The authors reviewed and edited all AI-assisted output and take full responsibility for the content of this publication.
