# Supplementary material for: Patient-Reported Adherence, Knowledge and Perceived Outcomes After Specialist Nursing Education in a Multidisciplinary Headache Unit: A Cross-Sectional Observational Study
Source: Nurs Rep. 2026 Oct 10;16(10):359. doi: 10.3390/nursrep16100359 (PMC13708254; doi:10.3390/nursrep16100359)
Supplement: Supplementary file 1 [file nursrep-16-00359-s001.zip › Supplementary File S1 STROBEchecklist.pdf]

Supplementary File S1

STROBE Statement — Checklist of items that should be included in reports of cross-sectional studies

Manuscript nursrep-4600767: "Patient-reported adherence, knowledge and perceived outcomes after specialist nursing education in a multidisciplinary headache unit: a cross-sectional observational study". Locations refer to the sections of the revised manuscript.

Section/Topic	Item No.	Recommendation	Reported in the revised manuscript
**Title and abstract**			
**Title and abstract**	1a	Indicate the study's design with a commonly used term in the title or the abstract	Title ("cross-sectional observational study"); Abstract, Methods
	1b	Provide in the abstract an informative and balanced summary of what was done and what was found	Abstract
**Introduction**			
**Background/rationale**	2	Explain the scientific background and rationale for the investigation being reported	Section 1 (Introduction), paragraphs 1–5
**Objectives**	3	State specific objectives, including any prespecified hypotheses	Section 1, last paragraph. No hypotheses were prespecified (descriptive study)
**Methods**			
**Study design**	4	Present key elements of study design early in the paper	Section 2.1 (single post-consultation survey; retrospective rating of prior knowledge; no control group)
**Setting**	5	Describe the setting, locations, and relevant dates, including periods of recruitment, exposure, follow-up, and data collection	Section 2.1 (Hospital Universitario La Paz, Madrid, Spain; January–December 2021); Section 2.3 (setting of the nursing consultation); Section 2.4 (survey sent by e-mail a median of 120 days after the consultation)
**Participants**	6a	Give the eligibility criteria, and the sources and methods of selection of participants	Section 2.2 (inclusion and exclusion criteria; consecutive recruitment of patients starting anti-CGRP monoclonal antibodies)
**Variables**	7	Clearly define all outcomes, exposures, predictors, potential confounders, and effect modifiers. Give diagnostic criteria, if applicable	Section 2.3 (exposure: nursing consultation); Section 2.5 (outcome definitions); Section 2.2 (ICHD-3 diagnosis); Section 3.1 and Table 1 (clinical variables)
**Data sources/measurement**	8*	For each variable of interest, give sources of data and details of methods of assessment (measurement). Describe comparability of assessment methods if there is more than one group	Section 2.4 (ad hoc questionnaire: development, expert review, pilot in 50 patients, 25 items, electronic administration); Section 2.5; Section 3.1 (clinical data from records); Supplementary File S2 (full questionnaire). Single group

Section/Topic	Item No.	Recommendation	Reported in the revised manuscript
**Bias**	9	Describe any efforts to address potential sources of bias	Section 2.2 (consecutive recruitment); Section 2.4 (survey completed at home, without the nurse present, to limit social desirability bias); Section 4.1 (recall, response-shift, selection and social-desirability bias)
**Study size**	10	Explain how the study size was arrived at	Section 2.2 (no formal calculation; all eligible patients who agreed during the 12-month period; precision of ±10 percentage points for proportions)
**Quantitative variables**	11	Explain how quantitative variables were handled in the analyses. If applicable, describe which groupings were chosen and why	Section 2.5 (four-point frequency scale, good adherence = “always”; agreement items analysed as yes/agree vs. no/disagree; adequate knowledge criterion); Section 2.7
**Statistical methods**	12a	Describe all statistical methods, including those used to control for confounding	Section 2.7 (descriptive statistics; 95% CIs by the Wilson score method). No adjustment for confounding (descriptive study)
	12b	Describe any methods used to examine subgroups and interactions	Not applicable (no subgroup analyses)
	12c	Explain how missing data were addressed	Section 2.7 (no missing data)
	12d	If applicable, describe analytical methods taking account of sampling strategy	Not applicable (consecutive sample)
	12e	Describe any sensitivity analyses	Not applicable (no sensitivity analyses)
**Results**			
**Participants**	13a*	Report numbers of individuals at each stage of study—e.g., numbers potentially eligible, examined for eligibility, confirmed eligible, included in the study, completing follow-up, and analysed	Section 2.2 (100 patients invited, 90 agreed to participate and were analysed); Section 3.1
	13b*	Give reasons for non-participation at each stage	Section 2.2 (10 invited patients declined participation)
	13c*	Consider use of a flow diagram	Not provided; participant flow described in the text (Section 2.2)
**Descriptive data**	14a*	Give characteristics of study participants (e.g., demographic, clinical, social) and information on exposures and potential confounders	Section 3.1; Table 1 (demographic and clinical characteristics, anti-CGRP agent, concomitant preventives, comorbidities, time to survey)
	14b*	Indicate number of participants with missing data for each variable of interest	Section 2.7 (no missing data)
**Outcome data**	15*	Report numbers of outcome events or summary measures	Sections 3.2–3.6; Figures 1–4

Section/Topic	Item No.	Recommendation	Reported in the revised manuscript
**Main results**	16a	Give unadjusted estimates and, if applicable, confounder-adjusted estimates and their precision (e.g., 95% confidence interval). Make clear which confounders were adjusted for and why they were included	Sections 3.2–3.4 (n, %, and 95% CI); Abstract, Results. Unadjusted estimates only
	16b	Report category boundaries when continuous variables were categorized	Section 2.5 (response categories); Figures 1–2
	16c	If relevant, consider translating estimates of relative risk into absolute risk for a meaningful time period	Not applicable
**Other analyses**	17	Report other analyses done—e.g., analyses of subgroups and interactions, and sensitivity analyses	Not applicable; barriers reported descriptively (Section 3.6)
**Discussion**			
**Key results**	18	Summarise key results with reference to study objectives	Section 4, first paragraph
**Limitations**	19	Discuss limitations of the study, taking into account sources of potential bias or imprecision. Discuss both direction and magnitude of any potential bias	Section 4.1 (design without control group; concurrent anti-CGRP initiation; retrospective knowledge rating, recall bias and response shift; variable survey timing; selection and social-desirability bias; non-validated instrument; single-item outcomes; sample size)
**Interpretation**	20	Give a cautious overall interpretation of results considering objectives, limitations, multiplicity of analyses, results from similar studies, and other relevant evidence	Section 4, paragraphs 1–8; Section 5 (Conclusions)
**Generalisability**	21	Discuss the generalisability (external validity) of the study results	Section 4.1 (single centre; patients starting anti-CGRP treatment with high-frequency migraine, 77.8% chronic and 78.9% with medication overuse)
**Other information**			
**Funding**	22	Give the source of funding and the role of the funders for the present study and, if applicable, for the original study on which the present article is based	Funding statement (back matter): no external funding

**Give information separately for exposed and unexposed groups, if applicable. This study included a single group.*

Checklist adapted from the STROBE Statement (von Elm E, et al. Lancet 2007;370:1453–1457). The STROBE checklist is available at www.strobe-statement.org.
