# Supplementary material for: Patient-Reported Adherence, Knowledge and Perceived Outcomes After Specialist Nursing Education in a Multidisciplinary Headache Unit: A Cross-Sectional Observational Study
Source: Nurs Rep. 2026 Oct 10;16(10):359. doi: 10.3390/nursrep16100359 (PMC13708254; doi:10.3390/nursrep16100359)
Supplement: Supplementary file 1 [file nursrep-16-00359-s001.zip › Supplementary File S2 Questionnaire.pdf]

**Supplementary File S2**

Nursing Reports — manuscript nursrep-4600767

*Study questionnaire: original Spanish version followed by the English translation. Section A (clinical data) was completed by the research team from the clinical records; items 1–25 were completed by patients electronically.*

# Cuestionario de educación en la consulta de enfermería de la Unidad de Cefaleas

*Versión original en español (25 ítems)*

Este cuestionario forma parte de un estudio sobre la consulta de enfermería de la Unidad de Cefaleas. Le llevará unos 10 minutos. Sus respuestas son confidenciales y solo se utilizarán con fines de investigación. No hay respuestas correctas ni incorrectas: conteste pensando en su experiencia real. Marque una sola casilla por pregunta, salvo cuando se indique lo contrario.

## Sección A. Datos sociodemográficos y clínicos (a cumplimentar por el equipo investigador a partir de la historia clínica)

| Variable | Dato |
|---|---|
| Código del paciente | ________ |
| Edad (años) | ____ |
| Sexo | ☐ Mujer ☐ Hombre |
| Tipo de migraña (ICHD-3) | ☐ Sin aura ☐ Con aura |
| Curso | ☐ Episódica ☐ Crónica |
| Años de evolución de la migraña | ____ |
| Días de cefalea al mes (último mes, según diario) | ____ |
| Abuso de medicación sintomática en la consulta | ☐ Sí ☐ No |
| Número de tratamientos preventivos previos (incluidos orales) | ____ |
| Anticuerpo anti-CGRP iniciado | ☐ Erenumab ☐ Galcanezumab ☐ Fremanezumab |
| Tratamiento preventivo oral concomitante | ☐ Sí ☐ No Cuál: ________ |
| OnabotulinumtoxinA concomitante | ☐ Sí ☐ No |
| Ansiedad y/o depresión | ☐ Sí ☐ No |
| Trastorno del sueño | ☐ Sí ☐ No |
| Otras comorbilidades | ________________ |
| Fecha de la consulta de enfermería | ___/___/______ |
| Fecha de cumplimentación del cuestionario | ___/___/______ |

## Sección B. Conocimientos sobre su migraña (ítems 1–5)

*Indique cuánto sabía ANTES de la consulta de enfermería y cuánto sabe AHORA sobre cada uno de los siguientes aspectos.*

| | ANTES de la consulta | | | | AHORA | | | |
|---|---|---|---|---|---|---|---|---|
| | Nada | Poco | Bastante | Mucho | Nada | Poco | Bastante | Mucho |
| 1. Identificar los desencadenantes de mis crisis | ☐ | ☐ | ☐ | ☐ | ☐ | ☐ | ☐ | ☐ |
| 2. Cómo y cuándo tomar la medicación para las crisis | ☐ | ☐ | ☐ | ☐ | ☐ | ☐ | ☐ | ☐ |
| 3. Qué cambios en el estilo de vida me ayudan a controlar la migraña | ☐ | ☐ | ☐ | ☐ | ☐ | ☐ | ☐ | ☐ |
| 4. Cómo rellenar correctamente el diario de cefalea | ☐ | ☐ | ☐ | ☐ | ☐ | ☐ | ☐ | ☐ |
| 5. Cómo evitar el abuso de medicación para el dolor | ☐ | ☐ | ☐ | ☐ | ☐ | ☐ | ☐ | ☐ |

## Sección C. Seguimiento de las recomendaciones (ítems 6–8)

*Desde la consulta de enfermería, ¿con qué frecuencia…*

| | Siempre | Habitualmente (>50% de las veces) | A veces (≤50% de las veces) | Nunca |
|---|---|---|---|---|
| 6. …sigue las recomendaciones de estilo de vida (sueño regular, buena hidratación, ejercicio aeróbico y manejo del estrés)? | ☐ | ☐ | ☐ | ☐ |
| 7. …toma la medicación para las crisis según las indicaciones de la enfermera (momento de la toma, dosis y evitar el abuso)? | ☐ | ☐ | ☐ | ☐ |
| 8. …rellena el diario de cefalea? | ☐ | ☐ | ☐ | ☐ |

## Sección D. Tratamiento con anticuerpo anti-CGRP (ítems 9–12)

*Indique su grado de acuerdo con las siguientes afirmaciones.*

| | Totalmente de acuerdo | De acuerdo | En desacuerdo | Totalmente en desacuerdo |
|---|---|---|---|---|
| 9. Sé administrarme correctamente el anticuerpo (técnica de inyección y rotación de zonas) | ☐ | ☐ | ☐ | ☐ |
| 10. Sé qué efectos adversos debo vigilar y cuándo consultar | ☐ | ☐ | ☐ | ☐ |
| 11. Sé cuánto tiempo puede tardar el tratamiento en hacer efecto | ☐ | ☐ | ☐ | ☐ |
| 12. Me administro el anticuerpo en las fechas indicadas | ☐ | ☐ | ☐ | ☐ |

## Sección E. Cambios percibidos (ítems 13–17)

| | Totalmente de acuerdo | De acuerdo | En desacuerdo | Totalmente en desacuerdo |
|---|---|---|---|---|
| 13. Desde que sigo los consejos de la enfermera, he notado que tengo menos crisis de migraña | ☐ | ☐ | ☐ | ☐ |

| | Totalmente de acuerdo | De acuerdo | En desacuerdo | Totalmente en desacuerdo |
|---|---|---|---|---|
| 14. Desde que sigo los consejos de la enfermera, he notado que mis crisis son menos intensas | ☐ | ☐ | ☐ | ☐ |
| 15. La educación recibida me ha ayudado a entender mejor mi migraña y a mejorar mi calidad de vida | ☐ | ☐ | ☐ | ☐ |
| 16. Me siento más seguro/a para manejar mi enfermedad | ☐ | ☐ | ☐ | ☐ |
| 17. Recomendaría esta consulta de enfermería a otros pacientes | ☐ | ☐ | ☐ | ☐ |

## Sección F. Valoración de la consulta (ítems 18–22)

| | Totalmente de acuerdo | De acuerdo | En desacuerdo | Totalmente en desacuerdo |
|---|---|---|---|---|
| 18. La información que recibí fue clara y comprensible | ☐ | ☐ | ☐ | ☐ |
| 19. El tiempo de la consulta fue suficiente | ☐ | ☐ | ☐ | ☐ |
| 20. Se resolvieron todas mis dudas | ☐ | ☐ | ☐ | ☐ |
| 21. La hoja de recomendaciones que me entregaron me resultó útil | ☐ | ☐ | ☐ | ☐ |
| 22. Sé cómo contactar con la Unidad si tengo dudas o problemas | ☐ | ☐ | ☐ | ☐ |

## Sección G. Dificultades, satisfacción y sugerencias (ítems 23–25)

23. ¿Qué le ha resultado más difícil a la hora de seguir las recomendaciones? (puede marcar varias opciones)

☐ Conocía poco la migraña antes de la consulta

☐ Me cuesta rellenar el diario de cefalea de forma continuada

☐ Falta de tiempo

☐ Olvidos

☐ Dificultad para cambiar mis hábitos (sueño, ejercicio, alimentación)

☐ Dudas o miedo sobre la medicación

☐ Otra: ________________________________

24. En una escala de 0 (nada satisfecho/a) a 10 (totalmente satisfecho/a), ¿cuál es su grado de satisfacción global con la consulta de enfermería?

| 0 | 1 | 2 | 3 | 4 | 5 | 6 | 7 | 8 | 9 | 10 |
|---|---|---|---|---|---|---|---|---|---|---|
| ☐ | ☐ | ☐ | ☐ | ☐ | ☐ | ☐ | ☐ | ☐ | ☐ | ☐ |

25. ¿Qué sugerencias haría para mejorar la consulta de enfermería? (respuesta libre)

______________________________________________

______________________________________________

______________________________________________

*Muchas gracias por su colaboración.*

# Headache Unit Nursing Consultation Education Questionnaire

*English translation (25 items). The questionnaire was administered in Spanish.*

This questionnaire is part of a study on the nursing consultation of the Headache Unit. It will take about 10 minutes. Your answers are confidential and will be used for research purposes only. There are no right or wrong answers: please answer based on your own experience. Tick one box per question unless otherwise indicated.

## Section A. Sociodemographic and clinical data (completed by the research team from the clinical records)

| Variable | Value |
|---|---|
| Patient code | ________ |
| Age (years) | ____ |
| Sex | ☐ Female ☐ Male |
| Migraine type (ICHD-3) | ☐ Without aura ☐ With aura |
| Course | ☐ Episodic ☐ Chronic |
| Disease duration (years) | ____ |
| Monthly headache days (last month, from diary) | ____ |
| Medication overuse at the consultation | ☐ Yes ☐ No |
| Number of previous preventive treatments (including oral) | ____ |
| Anti-CGRP monoclonal antibody started | ☐ Erenumab ☐ Galcanezumab ☐ Fremanezumab |
| Concomitant oral preventive treatment | ☐ Yes ☐ No Which: ________ |
| Concomitant onabotulinumtoxinA | ☐ Yes ☐ No |
| Anxiety and/or depression | ☐ Yes ☐ No |
| Sleep disorder | ☐ Yes ☐ No |
| Other comorbidities | ________________ |
| Date of nursing consultation | ___/___/______ |
| Date of questionnaire completion | ___/___/______ |

## Section B. Knowledge about your migraine (items 1–5)

*Please indicate how much you knew BEFORE the nursing consultation and how much you know NOW about each of the following.*

| | BEFORE the consultation | | | | NOW | | | |
|---|---|---|---|---|---|---|---|---|
| | None | A little | Quite a lot | A lot | None | A little | Quite a lot | A lot |
| 1. Identifying the triggers of my attacks | ☐ | ☐ | ☐ | ☐ | ☐ | ☐ | ☐ | ☐ |
| 2. How and when to take medication for my attacks | ☐ | ☐ | ☐ | ☐ | ☐ | ☐ | ☐ | ☐ |

| | BEFORE the consultation | | | | NOW | | | |
|---|---|---|---|---|---|---|---|---|
| | None | A little | Quite a lot | A lot | None | A little | Quite a lot | A lot |
| 3. Which lifestyle changes help me control my migraine | ☐ | ☐ | ☐ | ☐ | ☐ | ☐ | ☐ | ☐ |
| 4. How to complete the headache diary correctly | ☐ | ☐ | ☐ | ☐ | ☐ | ☐ | ☐ | ☐ |
| 5. How to avoid overusing pain medication | ☐ | ☐ | ☐ | ☐ | ☐ | ☐ | ☐ | ☐ |

## Section C. Following the recommendations (items 6–8)

*Since the nursing consultation, how often do you…*

| | Always | Usually (>50% of the time) | Sometimes (≤50% of the time) | Never |
|---|---|---|---|---|
| 6. …follow the lifestyle recommendations (regular sleep, adequate hydration, aerobic exercise, and stress management)? | ☐ | ☐ | ☐ | ☐ |
| 7. …take medication for your attacks as instructed by the nurse (timing, dose, and avoiding overuse)? | ☐ | ☐ | ☐ | ☐ |
| 8. …complete the headache diary? | ☐ | ☐ | ☐ | ☐ |

## Section D. Anti-CGRP monoclonal antibody treatment (items 9–12)

*Please indicate how much you agree with the following statements.*

| | Strongly agree | Agree | Disagree | Strongly disagree |
|---|---|---|---|---|
| 9. I know how to self-administer the antibody correctly (injection technique and site rotation) | ☐ | ☐ | ☐ | ☐ |
| 10. I know which adverse effects to watch for and when to seek advice | ☐ | ☐ | ☐ | ☐ |
| 11. I know how long the treatment may take to work | ☐ | ☐ | ☐ | ☐ |
| 12. I administer the antibody on the scheduled dates | ☐ | ☐ | ☐ | ☐ |

## Section E. Perceived changes (items 13–17)

| | Strongly agree | Agree | Disagree | Strongly disagree |
|---|---|---|---|---|
| 13. Since following the nurse's advice, I have noticed fewer migraine attacks | ☐ | ☐ | ☐ | ☐ |
| 14. Since following the nurse's advice, I have noticed that my attacks are less intense | ☐ | ☐ | ☐ | ☐ |
| 15. The education I received has helped me understand my migraine better and improve my quality of life | ☐ | ☐ | ☐ | ☐ |
| 16. I feel more confident managing my condition | ☐ | ☐ | ☐ | ☐ |

| | Strongly agree | Agree | Disagree | Strongly disagree |
|---|---|---|---|---|
| 17. I would recommend this nursing consultation to other patients | ☐ | ☐ | ☐ | ☐ |

## Section F. Assessment of the consultation (items 18–22)

| | Strongly agree | Agree | Disagree | Strongly disagree |
|---|---|---|---|---|
| 18. The information I received was clear and easy to understand | ☐ | ☐ | ☐ | ☐ |
| 19. The consultation lasted long enough | ☐ | ☐ | ☐ | ☐ |
| 20. All my questions were answered | ☐ | ☐ | ☐ | ☐ |
| 21. The written recommendations sheet I received was useful | ☐ | ☐ | ☐ | ☐ |
| 22. I know how to contact the Unit if I have questions or problems | ☐ | ☐ | ☐ | ☐ |

## Section G. Difficulties, satisfaction, and suggestions (items 23–25)

23. What has been most difficult for you when following the recommendations? (you may tick more than one option)

☐ I knew little about migraine before the consultation

☐ I find it hard to keep up the headache diary

☐ Lack of time

☐ Forgetting

☐ Difficulty changing my habits (sleep, exercise, diet)

☐ Doubts or fears about the medication

☐ Other: ________________________________

24. On a scale from 0 (not at all satisfied) to 10 (completely satisfied), how satisfied are you overall with the nursing consultation?

| 0 | 1 | 2 | 3 | 4 | 5 | 6 | 7 | 8 | 9 | 10 |
|---|---|---|---|---|---|---|---|---|---|---|
| ☐ | ☐ | ☐ | ☐ | ☐ | ☐ | ☐ | ☐ | ☐ | ☐ | ☐ |

25. What suggestions would you make to improve the nursing consultation? (free text)

________________________________________________________________

________________________________________________________________

________________________________________________________________

________________________________________________________________

*Thank you very much for your participation.*
